# Cell Signaling Pathway in 12-O-Tetradecanoylphorbol-13-acetate-Induced LCN2 Gene Transcription in Esophageal Squamous Cell Carcinoma

**DOI:** 10.1155/2017/9592501

**Published:** 2017-10-02

**Authors:** Lingying Meng, Muting Wang, Zepeng Du, Zhongmin Fang, Bingli Wu, Jianyi Wu, Wenming Xie, Jian Shen, Tianxiang Zhu, XieE Xu, Liandi Liao, Liyan Xu, Enmin Li, Bin Lan

**Affiliations:** ^1^Department of Cardiothoracic Surgery, Shantou Central Hospital, Affiliated Shantou Hospital of Sun Yat-sen University, Shantou, Guangdong 515041, China; ^2^Department of Pathology, Shantou Central Hospital, Affiliated Shantou Hospital of Sun Yat-sen University, Shantou, Guangdong 515041, China; ^3^Department of Biochemistry and Molecular Biology, Shantou University Medical College, Shantou, Guangdong 515041, China; ^4^Network and Information Center, Shantou University Medical College, Shantou 515041, China; ^5^Department of Oncologic Pathology, Shantou University Medical College, Shantou, Guangdong 515041, China

## Abstract

LCN2 is involved in various cellular functions, including transport of small hydrophobic molecules, protection of MMP9 from proteolytic degradation, and regulating innate immunity. LCN2 is elevated in multiple human cancers, frequently being associated with tumor size, stage, and invasiveness. Our previous studies have shown that LCN2 expression could be induced by 12-O-tetradecanoylphorbol-13-acetate (TPA) in esophageal squamous cell carcinoma (ESCC) by the binding of five nucleoproteins (MISP, KLF10, KLF15, PPP1R18, and RXR*β*) at a novel TPA-responsive element (TRE), at −152~−60 bp of the 5′ flanking region of the* LCN2* promoter. However, much is unknown about whether these proteins can respond to TPA stimulation to regulate LCN2 transactivation and which cell signaling pathways mediate this process. In this study, expression plasmids encoding these five nucleoproteins were stably transfected into EC109 cells. Then, stable transfectant was characterized by a Dual-Luciferase Reporter Assay System. RT-PCR, real-time PCR, western blotting, specific kinase inhibitor treatment, and bioinformatics analyses were applied in this study. We found that MISP, KLF10, KLF15, PPP1R18, and RXR*β* proteins could strongly respond to TPA stimulation and activate LCN2 transcriptional expression. MEK, ERK, JNK, and P38 kinases were involved in the LCN2 transactivation. Furthermore, the MEK-ERK signal pathway plays a major role in this biological process but does not involve PKC*α* signaling.

## 1. Introduction

Lipocalin 2 (LCN2), also named neutrophil gelatinase-2 associated lipocalin (NGAL), a member of the lipocalin family, was originally found in granules from human neutrophils [1]. LCN2 is involved in various cellular functions, such as transport of small hydrophobic molecules and protection of MMP9 from proteolytic degradation. LCN2 tightly binds to bacterial siderophores, serving as a potent bacteriostatic agent by sequestering iron and regulating innate immunity [2, 3]. Elevated LCN2 expression has also been observed in multiple human cancers including breast, colorectal, pancreatic, ovarian, gastric, thyroid, ovarian, bladder, and kidney cancers, as well as glioma and esophageal squamous cell carcinoma (ESCC) [4–10]. LCN2 is frequently associated with tumor size, stage, and invasiveness, involving in the invasion and poor prognosis of carcinoma cells. These features characterize LCN2 as a potential biomarker in malignancy.

However, the molecular mechanism underlying the upregulation of LCN2 in tumor cells has not been fully illustrated. Altered LCN2 expression in diseases has led investigators to examine the mechanisms of its transcriptional regulation. The expression of LCN2 can be induced by various means, such as LPS, oxidative stress, metabolic stress, cytokines, and nutrients [11–13]. Increasing evidence indicates that transcription factors, such as IkBz, NF-kB, and ELF3 (E74-like factor 3), play crucial roles in the regulation of LCN2 expression in tumor cells of various origins, including lung and chondrocytes [14, 15]. MUC4 regulates LCN2 by stabilizing HER2 and stimulating AKT, which results in the activation of NF-*κ*B in pancreatic cancer [16]. Knockdown experiments also demonstrated that STAT1 is required for IFN*γ*-induced LCN2 expression in murine adipocytes [17]. These studies indicate that distinctive regulatory elements or mechanisms contribute to the induced expression of LCN2 in different cell types responding to various factors.

Our previous work revealed that LCN2 is significantly increased in ESCC and can serve as a marker for poor prognosis [6]. LCN2 promotes the migration and invasion of ESCC cells through a novel positive feedback loop [18]. Moreover, we found LCN2 could be induced by the tumor-promoting agent 12-O-tetradecanoylphorbol-13-acetate (TPA), in esophageal cancer cells at the transcriptional level. A TPA-responsive element (TRE) is located at −152 to −60 from the 5′-flanking region of the* LCN2* promoter. Several nucleoproteins (MISP, KLF10, KLF15, PPP1R18, and RXR*β*) have been identified by affinity chromatography and mass spectrometry and could respond to TPA stimulation and regulate LCN2 expression in esophageal cancer cells [19, 20]. However, the transcriptional regulation mechanism of LCN2 by TPA in ESCC has not been reported. This study aims to further investigate the signal pathways involved in TPA-induced LCN2 gene transcriptional regulation mediated by MISP, KLF10, KLF15, PPP1R18, and RXR*β*.

## 2. Materials and Methods

### 2.1. Cell Lines, Plasmids, and Reagents

The ESCC cell line EC109 was cultured in 199 medium (Invitrogen, Carlsbad, USA) containing 10% (v/v) fetal bovine serum and maintained at 37°C in a humidified 5% CO_2_ atmosphere. TPA was purchased from Sigma-Aldrich (St. Louis, MO, USA). DMSO was purchased from Amresco Company (Solon, Ohio, USA). Empty pcDNA3 vector was purchased from Invitrogen. PKC*α*/*β* kinase inhibitors (myristoylated protein kinase C peptide inhibitor), MEK kinase inhibitors (U0126, PD98059), and p38 kinase inhibitors SB203580 were purchased from Promega (Madison, WI, USA). The c-Jun N-terminal kinase (JNK) inhibitor SP600125 was purchased from Calbiochem (La Jolla, CA, USA). Antibodies against phospho-ERK1/2 (p-ERK1/2), ERK1/2, and JNK were purchased from Santa Cruz Biotechnology (Santa Cruz, USA). *β*-Actin was purchased from Sigma-Aldrich (St. Louis, MO, USA). The pMD19-T Simple vector, SYBR Premix Ex-Taq™, and SYBR Primescript RT-PCR Kit were purchased from TaKaRa (Dalian, China).

### 2.2. Expression Vector Construction and Stable Transfection

The full-length cDNAs for MISP, KLF10, KLF15, PPP1R18, and RXR*β* were amplified by RT-PCR. The primer sequences and enzyme restriction sites are listed in Table 1. PCR products were gel purified and initially cloned into the pMD19-T Simple vector. The sequences were confirmed by DNA sequencing and were directly inserted into the pcDNA3.0 vector, resulting in the production of pc-MISP, pc-KLF10, pc-KLF15, PPP1R18, and pc-RXR*β*. EC109 cells were inoculated into 6-well plates, grown to 50–80% confluence and transfected with 2 *μ*g of each of the five expression plasmids, using pcDNA3.0 as the control, with Superfect Transfection Reagent (QIAGEN, Hilden, Germany) according to the manufacturer's instructions. After transfection, cells were incubated for 48 h and selected in 400 mg/L G418 for one month, until single clones arose, after which the G418 concentration was reduced to 200 mg/L in the growth medium. EC109 clones with high expression of MISP, KLF10, KLF15, PPP1R18, and RXR*β* were confirmed by RT-PCR.

### 2.3. Dual-Luciferase Reporter Assays Analyses

Luciferase reporter plasmid pGLB152 containing the LCN2 gene 5′-flanking region of −152 to +84 bp, constructed in our laboratory, is described in a previous report [19]. Plasmid pGL3-Basic (pGLB) contains a modified coding region for firefly* (Photinus pyralis)* luciferase that has been optimized for monitoring transcriptional activity in transfected eukaryotic cells. Plasmid pRLTK (Promega), containing a cDNA* (Rluc)* encoding* Renilla* luciferase originally cloned from the marine organism* Renilla reniformis*, was used as an internal control for transfection efficiency in dual-luciferase reporter assays. EC109 cells were cotransfected with pGLB152 and pcDNA3.0 recombinant expression vectors. Briefly, EC109 cells were inoculated into a 96-well plate at 0.8~1.0 × 10^5^ cells/ml, grown to 50–80% confluency, and cotransfected with 0.3 *μ*g recombinant expression vector (pc-MISP, pc-KLF10, pc-KLF15, PPP1R18, and pc-RXR*β*), 0.3 *μ*g plasmid pGLB152, and 0.012 *μ*g of pRLTK, using Superfect Transfection Reagent (QIAGEN, Hilden, Germany) according to the manufacturer's instructions. Stably transfected EC109 cell lines with high expression of MISP, KLF10, KLF15, PPP1R18, and RXR*β* were transfected with 0.5 *μ*g pGLB152 and 0.01 *μ*g pRLTK. After the transfection, cells were incubated for 48 h and harvested in Passive Lysis Buffer (Promega). The purpose of the two experiments described above was to validate the effects of MISP, KLF10, KLF15, PPP1R18, and RXR*β* overexpression on LCN2 gene promoter activity.

In another luciferase assay, EC109 cells were transfected with 0.5 *μ*g pB152 and 0.01 *μ*g pRLTK. At 24 h after transfection with the reporter, cells were pretreated with different kinase inhibitors (U0126, PD98059, SB203580, and SP600125) in a series of doses. To analyze the effect of TPA on luciferase reporter activity, after the transfection with the reporters for 24 h, transfected cells were treated with TPA (5 ng/mL) for an additional 24 h before harvest. All experiments were repeated at least 2 times in triplicate. All luciferase reporter activities were measured using the Dual-Luciferase Reporter Assay System (Promega) according to the manufacturer's recommendations.

### 2.4. Western Blot Analyses

EC109 cells were exposed to two different treatments, one where cells were treated with 5 ng/ml TPA for 0 h, 3 h, 6 h, 12 h, and 24 h and another where EC109 cells were pretreated with different doses of kinase inhibitors for 1 hour and then treated with 5 ng/ml TPA for 6 h, 12 h, and 24 h. The whole cell protein was extracted in by radioimmunoprecipitation assay (RIPA) buffer [50 mM Tris HCl, pH 8.0, 150 mM NaCl, 1% (vol/vol) Nonidet P-40, 0.5% (wt/vol) sodium desoxycholate, 0.1% (wt/vol) SDS] containing a complete protease inhibitor cocktail (Santa Cruz); then western blot analyses were performed with the following primary antibodies: mouse anti-p-ERK1/2, rabbit anti-ERK1/2 rabbit anti-phosphorylated JNK (p-JNK), and mouse anti-*β*-actin. All western blot experiments were repeated 2-3 times.

### 2.5. Real-Time Quantitative-PCR

Total cellular RNA was extracted with TRIzol from EC109 cells pretreated with MEK inhibitors (10 *μ*M of U0126, 15 *μ*M of PD98059) for 1 h and subsequently induced with 5 ng/ml TPA for another 24 h. The total RNA was reverse-transcribed to cDNA using a PrimeScript™ RT-PCR kit. The real-time RT-PCR assay was carried out with a Rotor-Gene 6000 system (Corbett Life Science, Sydney, Australia) using SYBR Premix Ex-Taq according to the manufacturer's instructions. Each PCR mixture contained 1 *μ*l cDNA, Premix Ex-Taq, and primer (0.2 *μ*mol/L) to a final volume of 10 *μ*l. Primer sequences for LCN2 and *β*-actin were as follows: LCN2-F: 5′-CCTCCCTGAAAACCACATCGT-3′, LCN2-R: 5′-TGTGCACTCAGCCGTCGATA-3′, *β*-actin-F: 5′-CAACTGGGACGACATGGAGAAA-3′, *β*-actin-R: 5′-GATAGCAACGTACATGGCTGGG-3′. All PCR reactions were performed in triplicate, and each experiment was repeated twice. The absolute expression level of LCN2 mRNA was normalized to that of *β*-actin mRNA.

### 2.6. Reverse Transcription-PCR

EC109 cells pretreated with different doses MEK specific inhibitors for 1 h and subsequently treated with 5 ng/ml TPA for another 24 h; then RNA was extracted and cDNA prepared. The mRNA levels of MISP, KLF10, KLF15, PPP1R18, and RXR*β* were determined by PCR amplification. Primers for PCR were as described previously [19]. Amplified products were separated on 1.5% agarose gels and visualized by FluorChem 8900 (Alpha Innotech, California, USA). GAPDH mRNA was used as an internal control.

### 2.7. Bioinformatics Analyses

The serine, threonine, and tyrosine phosphorylation sites in MISP, KLF10, KLF15, PPP1R18, and RXR*β* proteins were analyzed by NetPhos 2.0 Serve (http://www.cbs.dtu.dk/services/NetPhos/). The potential phosphorylation was determined to be higher than 0.996.

### 2.8. Statistical Analysis

The significance of differences between groups was calculated using the independent sample *t*-test. All statistical tests were performed with SPSS 13.0 (SPSS, Inc., Chicago, IL). Differences were considered statistically significant if *P* ≤ 0.05.

## 3. Results

### 3.1. MISP, KLF10, KLF15, PPP1R18, and RXR*β* Upregulate LCN2 Promoter Activity and mRNA Expression

**Figure 1:**
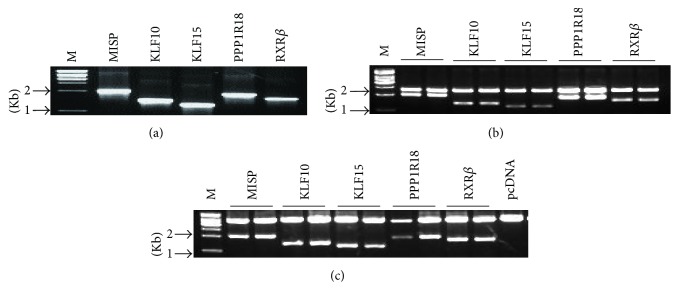
Identification of RT-PCR (Reverse Transcription-Polymerase Chain Reaction) products and recons MISP, KLF10, KLF15, PPP1R18, and RXR*β*. (a) Clone of full coding sequences of MISP, KLF10, KLF15, PPP1R18, and RXR*β* by RT-PCR from EC109. (b) Identification of pMD19-T vector recombinants by double restriction enzyme digestion. (c) Identification of pcDNA3 recombinant plasmids by restriction enzyme digestion.

**Figure 2:**
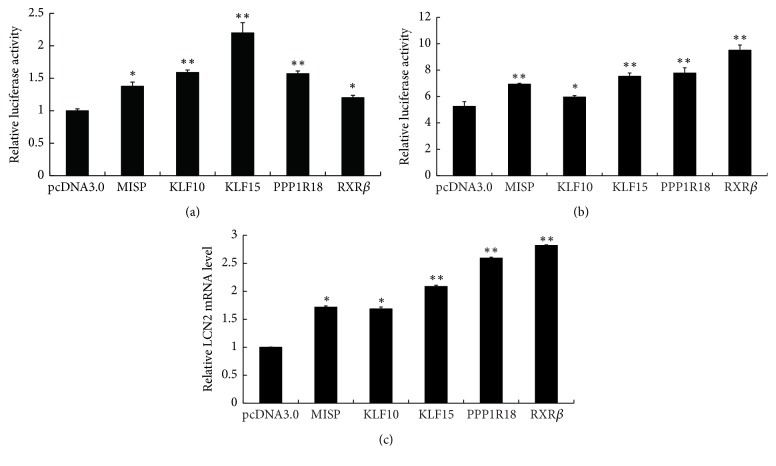
Effect of MISP, KLF10, KLF15, PPP1R18, and RXR*β* overexpression on the promoter activity and mRNA level of* LCN2* gene. (a) Transient cotransfection analysis of* LCN2* gene promoter activity. pB152 and pcDNA3 recombinants containing the five genes were cotransfected into EC109 cells, respectively. Luciferase activities of pB152 were normalized to* Renilla* luciferase activity and then shown relative to that of pcDNA3 control, which was set as 1. Each value represents the mean ± SD (standard deviation). The data were representative of at least two independent experiments. Transfections were carried out in triplicate for each experiment. (b) Relative luciferase activity analysis of pB152 in EC109 cells with stable expression of MISP, KLF10, KLF15, PPP1R18, and RXR*β*. (c) Analyses of LCN2 mRNA level in cells with stable expression of MISP, KLF10, KLF15, PPP1R18, and RXR*β* by real-time PCR. *β*-Actin was applied as an internal control. ^*∗*^*P* < 0.05, ^*∗∗*^*P* < 0.01.

We constructed a series of expression vectors (pc-MISP, pc-KLF10, pc-KLF15, pc-PPP1R18, and pc-RXR*β*) and transfected them into EC109 cells to acquire stably transfected cell lines following G418 selection (Figure 1). To confirm whether these five nucleoproteins were involved in the expression of LCN2, the recombinant expression vectors and pGLB152 were transiently cotransfected into EC109 cells. The reporter activities for LCN2 expression were significantly increased (Figure 2(a)). This was also observed in EC109 cells stably expressing each of the five nucleoproteins upon cotransfection with pGLB152 (Figure 2(b)). Quantitation of LCN2 mRNA levels by real-time PCR showed a similar increase LCN2 mRNA levels by overexpression of each protein (Figure 2(c)). These results indicate that nucleoproteins MISP, KLF10, KLF15, PPP1R18, and RXR*β* upregulate LCN2 gene expression at the transcription level and that LCN2 could be a target gene of these nucleoproteins.

### 3.2. MEK/ERK1/2 Signaling Pathway Is Involved in TPA-Induced* LCN2* Gene Transcription

**Figure 3:**
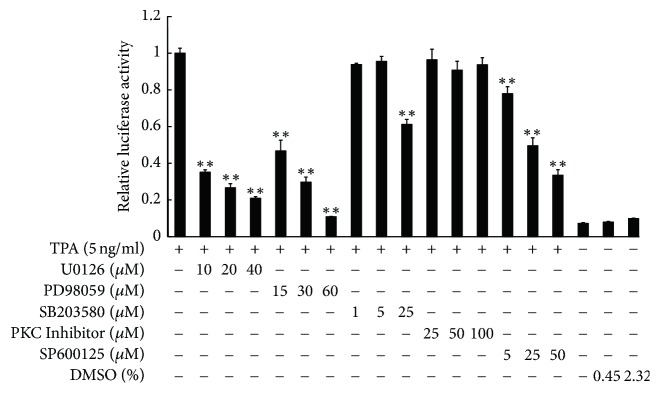
Effect of kinase specific inhibitors on the TPA-responsive ability of the* LCN2* gene promoter. EC109 cells were transfected with plasmid pB152 for 24 h and pretreated with various specific inhibitors (U0126, PD98059, SB203580, PKC inhibitor, and SP600125) for another 1 h; then TPA was added to treat 24 h before harvesting the cells. Data analysis was performed using SPSS 13.0. The independent sample *t*-test statistical method was used to determine the significance of differences between TPA treating control group and each TPA and specific kinase inhibitors experiment group. Differences were considered statistically significant at *P* ≤ 0.05. ^*∗∗*^*P* < 0.01.

**Figure 4:**
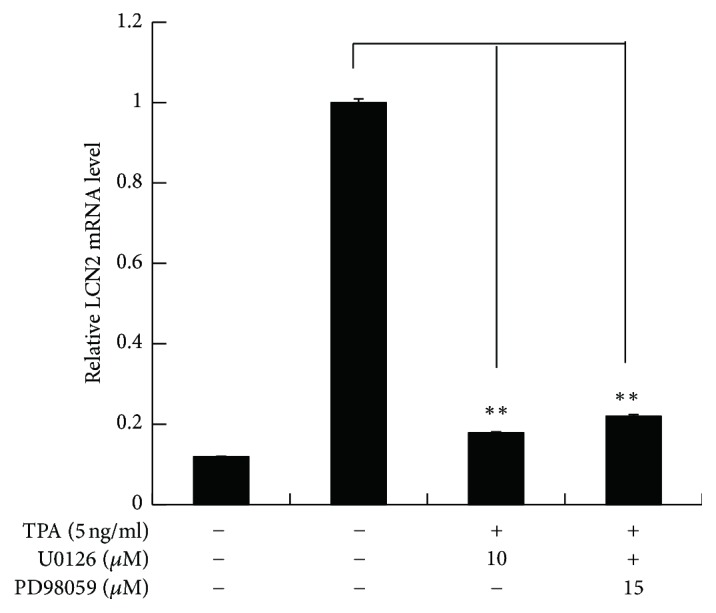
Analyses of* LCN2* mRNA levels in EC109 treated with MEK (mitogen-activated protein kinase/ERK kinase) specific inhibitors. Total RNA was extracted from EC109 cells treated with MEK specific inhibitors U0126 and PD98059, respectively. Real-time PCR assay was carried out with the Rotor-Gene 6000 system. All PCRs were performed in triplicate. The relative LCN2 mRNAs levels were normalized to that of *β*-actin mRNA and then shown to be relative to that of TPA treating control group. Differences were considered statistically significant at *P* ≤ 0.05 (^*∗∗*^*P* < 0.01).

To explore which signal transduction pathways are activated in TPA-induced LCN2 transcription, EC109 cells were cotransfected with constructs pB152 and pRLTK. At 24 h following transfection, cells were first pretreated with the following kinase inhibitors, MEK1/2 inhibitor (U0126 and PD98059), p38 MAPK inhibitor (SB203580), JNK inhibitor (SP600125), or PKC*α*/*β* inhibitor (myristoylated protein kinase C peptide inhibitor), and then induced by 5 ng/ml TPA for an additional 24 h, and the activity of the LCN2 promoter was assessed by luciferase reporter assays. The activity of the* LCN2* promoter following TPA treatment was at least 12 times than that of the nontreated control. MEK1/2 inhibitor (U0126 and PD98059) and JNK inhibitor (SP600125) significantly inhibited* LCN2* reporter gene induction in a dose-dependent manner (*P* < 0.01). MEK inhibitors was more effective than JNK inhibitor, with nearly 90% activity being inhibited by PD98059 at 60 *μ*M. The p38 MAPK inhibitor had no effect on* LCN2* gene promoter activity at low concentrations (1 *μ*M or 5 *μ*M), but LCN2 promoter activity was inhibited by 40% (*P* < 0.01) at concentrations up to 25 *μ*M. Nevertheless, PKC*α*/*β* kinase inhibitors showed no effect (Figure 3). Next, we investigated which signaling pathways were activated during the TPA induction by analyzing LCN2 mRNA levels by real-time PCR following pretreatment of EC109 cells with MEK inhibitors for 1 h. LCN2 mRNA levels were decreased by more than 80% on average, compared with the TPA-induced group, close to the nontreated control (*P* < 0.01) (Figure 4).

**Figure 5:**
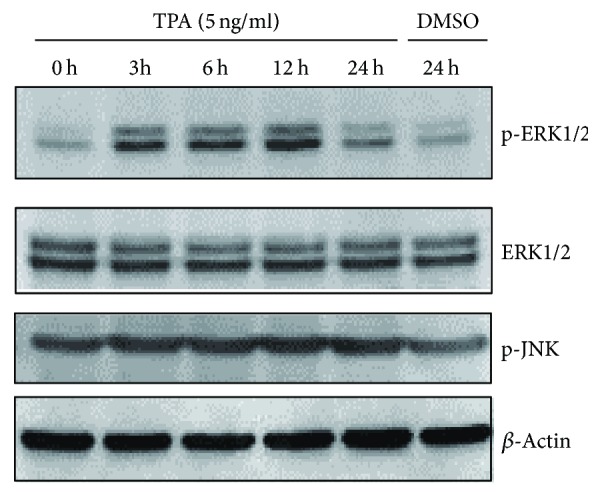
The analyses of MAPK (mitogen-activated protein kinase) signal pathway proteins in TPA-treated EC109 cells. Total protein from EC109 cells treated with TPA for 0 h, 3 h, 6 h, 12 h, and 24 h was obtained; then ERK1/2 (extracellular regulated protein kinases 1 and 2), p-ERK1/2 (phospho-ERK1/2), and p-JNK (phospho- c-jun N-terminal kinase) were detected by western blotting, respectively. *β*-Actin was shown as a loading control.

**Figure 6:**
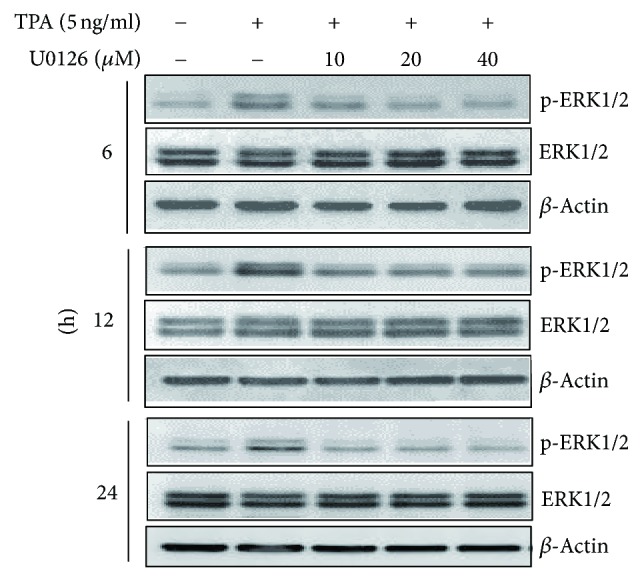
The analyses of ERK1/2 and p-ERK1/2 in EC109 cells treated with MEK specific inhibitor U0126. Total protein was collected from EC109 cells pretreated with MEK specific inhibitor (U0126) for 1 h and then treated with TPA for another 6 h, 12 h, and 24 h. ERK1/2 and p-ERK1/2 detection were, respectively, detected by western blotting.

Following induction of EC109 cells with TPA, the level of p-ERK1/2, peaked at 12 h and then decreased by 24 h, but not ERK1/2, p-JNK, and p-38. This pattern was similar to TPA-induced LCN2 gene expression in EC109 cells (Figure 5). Moreover, TPA-induced p-ERK1/2 levels could be blocked by MEK inhibitor U0126 in both a dose-dependent and time-dependent manner (Figure 6). These findings suggest that transcription of the* LCN2* gene is induced by TPA, and the increase of* LCN2* gene promoter activity following TPA induction is mainly mediated by the MEK-ERK signal pathway, but not the JNK or p38 MAPKs, or the previously reported PKC*α*/*β* signal pathway. Therefore, p-ERK1/2 plays a major role in TPA-mediated* LCN2* gene induction.

### 3.3. Effects of MEK Inhibitors on Transcriptional Activation by MISP, KLF10, KLF15, PPP1R18, and RXR*β*

**Figure 7:**
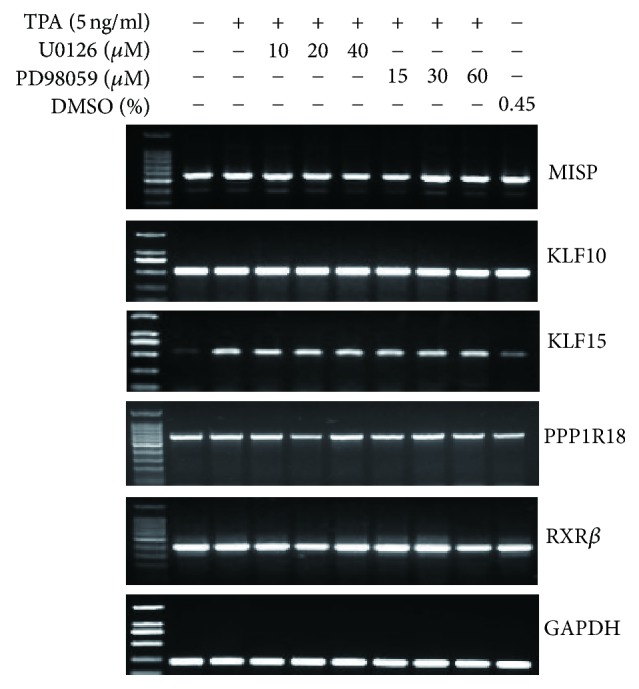
The expression level of MISP, KLF10, KLF15, PPP1R18, and RXR*β* in EC109 cells treated with MEK specific inhibitor. Total RNA was extracted from EC109 cells pretreated with MEK specific inhibitors (U0126 and PD98059) for 1 h and then treated with TPA for another 24 h. RT-PCR was performed to analyze the mRNA expression of MISP, KLF10, KLF15, PPP1R18, and RXR*β*, with GAPDH shown as an internal control.

Our previous study showed that mRNA levels of MISP, KLF10, KLF15, PPP1R18, and RXR*β* could be induced by TPA, indicating they could be TPA-responsive element- (TRE-) binding proteins [19]. In order to reveal whether the expressions of these new TRE-binding proteins were regulated by the MEK-ERK signal pathway, we detected the mRNA levels of the above genes in EC109 cells treated with MEK inhibitors. The results showed that the TPA-induced transcription of these genes is not inhibited by MEK inhibitors (Figure 7).

Next, to reveal the potential phosphorylation modification of these five nucleoproteins by p-ERK1/2, we analyzed the serine, threonine, and tyrosine phosphorylation sites in MISP, KLF10, KLF15, PPP1R18, and RXR*β* protein sequences by using the NetPhos 2.0 Server. We found there were 19 potential phosphorylation sites, for example, T-287, S-460, S-600, and S-675 in MISP, S-72, S-97, and S-121 in KLF10, S-40 in KLF15, S-125, S-133, S-139, S-145, S-175, S-213, S-224, S-245, and S-248 in PPP1R18, and S-76 and S-331 in RXR*β*. This indicates that the MEK-ERK signal pathway likely phosphorylates the above proteins in TPA-induced LCN2 expression.

## 4. Discussion

**Figure 8:**
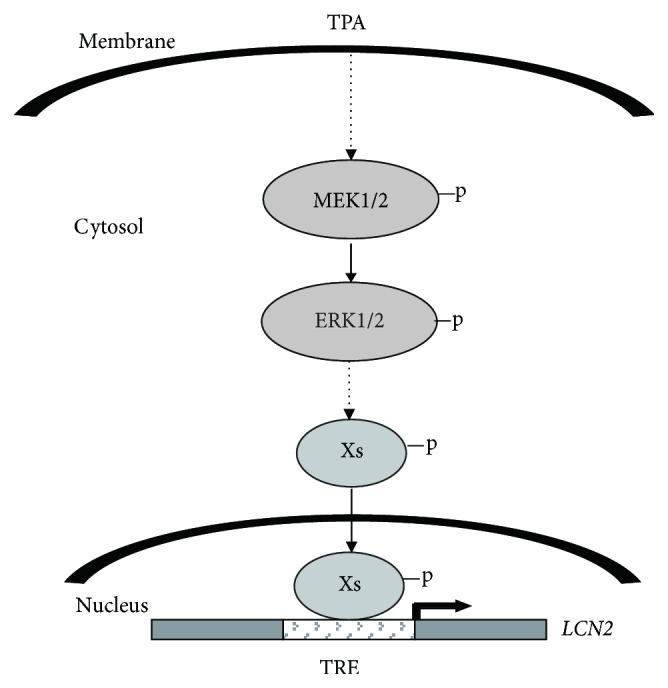
Schematic presentation of proposed signal pathway for regulating* LCN2* gene expression in esophageal carcinoma cells. Xs, transcription factors bound to TPA-responsive element, such as MISP, KLF10, KLF15, PPP1R18, and RXR*β*. TRE, TPA-responsive element.

TPA has been long used as a carcinogenesis promoting agent in various models [21]. Our previous study found that TPA could induce immortalization of esophageal epithelial cells (SHEEs) into cancer cells (SHEECs) [22]. During this progression, the* LCN2* gene is upregulated, indicating the LCN2 expression could be induced by TPA [18]. We further showed that* LCN2* gene could be induced by TPA and might contain a novel TPA-responsive element (TRE) in −152~−60 bp of its promoter. Five nucleoproteins MISP, KLF10, KLF15, PPP1R18, and RXR*β* were identified by oligonucleotide trapping, as the binding factors on the TRE under TPA stimulation, and were expressed at varying expression levels in esophageal cancer [19, 20]. However, the signaling pathways that participate in these events are poorly understood. In this study, we identify the signal transduction pathway of TPA-induced LCN2 expression in esophageal cancer. MISP, KLF10, KLF15, PPP1R18, and RXR*β* respond to TPA stimulation and enhance the transcriptional expression of* LCN2*. MEK → ERK, JNK, and p38 were found to regulate the TPA-induced* LCN2* expression, especially the MEK → ERK pathway. The previously reported TPA-PKC*α*/*β* does not appear to be involved in this process. Our results lead to a model involving MEK → ERK as being the major signaling pathway in TPA-induced LCN2 expression, and MISP, KLF10, KLF15, PPP1R18, and RXR*β* are the nuclear transcription factors that respond to TPA-mediated regulation of LCN2 transcription (Figure 8).

Several studies report that the effect of TPA is dependent on PKC*α* activation in tumor cells [23–25]. In contrast, PKC*α* does not play an important role in our study. This could be due to different TPA activation mechanisms between esophageal cancer cells and other types of cancer cells. Chien et al. found TPA-induced invasion and migration of HepG2 cells through a protein kinase C/extracellular signal-regulated kinase (PKC/ERK) pathway [25]. Similarly, it was found by Cheng et al. that calycosin could suppress lung cancer A549 cell proliferation and metastasis induced by TPA via inhibition of the PKC-*α*/ERK1/2 pathway [26]. Under these conditions, MEK activation and ERK phosphorylation led to the upregulation of downstream genes that bind to the* LCN2* promoter following addition of TPA, although PKC*α*/PKC*β* inhibition did not affect TPA-mediated LCN2 induction. We assume there are other TPA-activated signaling molecules that can stimulate MEK → ERK in ESCC cells.

The relationship between the MEK-ERK pathway and MISP, KLF10, KLF15, PPP1R18, and RXR*β* has not been delineated, but homologous proteins have been shown to be involved. Shimizu et al. have found that phosphorylated RXR*α* is phosphorylated by p-ERK1/2, a critical mechanism in the development of hepatocellular carcinoma [27]. Thus, it is possible that RXR*β* might also be the potential phosphorylation target of p-ERK1/2 in ESCC cells. Based on computer analyses, dozens of potential ERK phosphorylation sites were found in these five nucleoproteins. We propose that phosphorylation modification of the five nucleoproteins by p-ERK1/2 plays an important role in the upregulation of LCN2 expression induced by TPA.

In conclusion, our previous studies have suggested that LCN2 plays a critical role and has a distinctive regulatory mechanism in esophageal cancer [19, 20]. This study shows that C19, KLF10, KLF15, KIAA1949, and RXR*β* proteins can strongly respond to TPA stimulation and activate LCN2 transcription; the MEK → ERK, JNK, and P38 kinases, but not PKC*α*, are involved in LCN2 transactivation, indicating cell-specific regulation of LCN2.

## Figures and Tables

**Table 1:** Primers for amplifying full coding sequences of selected genes.

Name	Sequence (5′ → 3′)	Restriction site	Length of product
MISP-F1 MISP-R1	CCCAAGCTTAGATGGACCGCGTGACCAGATA CGGGATCCCCATCCCGAGGCTCAGTCAT	*Hin*dIII *Bam*HI	2054 bp
KLF10-F1 KLF10-R1	CCCAAGCTTATGCTCAACTTCGGTGCCTCT CGGGATCCTCTTCACTTTCCGGTCTGTC	*Hin*dIII *Bam*HI	1461 bp
KLF15-F1 KLF15-R1	CCCAAGCTTCCAGCATGGTGGACCACTTAC CGGGATCCGGTTCAGGGCGCTTTCAGTT	*Hin*dIII *Bam*HI	1270 bp
PPP1R18-F1 PPP1R18-R1	GGAATTCCCTACCCTCACCTCAAGACG GCTCTAGACCCTATGTTGGAAGATGGTCA	*Eco*RI *Xba*I	1883 bp
RXR*β*-F1 RXR*β*-R1	CCCAAGCTTCAGGGATCATGTCTTGGG GGAATTCGAGAAGCACCACGTCTGGGT	*Hin*dIII *Eco*RI	1636 bp
